# p73 expression is regulated by ribosomal protein RPL26 through mRNA translation and protein stability

**DOI:** 10.18632/oncotarget.13126

**Published:** 2016-11-04

**Authors:** Min Zhang, Jin Zhang, Wensheng Yan, Xinbin Chen

**Affiliations:** ^1^ College of Life Sciences and Technology, Huazhong Agricultural University, Wuhan, China; ^2^ Comparative Oncology Laboratory, Schools of Veterinary Medicine and Medicine, University of California at Davis, Davis, CA, USA

**Keywords:** RPL26, p73, MDM2, eIF4E, protein stability

## Abstract

p73, a p53 family tumor suppressor, is regulated by multiple mechanisms, including transcription and mRNA and protein stability. However, whether p73 expression is regulated via mRNA translation has not been explored. To test this, we examined whether ribosomal protein 26 (RPL26) plays a role in p73 expression. Here, we showed that p73 expression is controlled by RPL26 via protein stability and mRNA translation. To examine whether MDM2 mediates RPL26 to regulate p73 protein stability, we generated multiple MDM2-knockout cell lines by CRISPR-cas9. We found that in the absence of MDM2, the half-life of p73 protein is markedly increased. Interestingly, we also found that RPL26 is still capable of regulating p73 expression, albeit to a lesser extent, in MDM2-KO cells compared to that in isogenic control cells, suggesting that RPL26 regulates p73 expression via multiple mechanisms. Indeed, we found that RPL26 is necessary for efficient assembly of polysomes on p73 mRNA and de novo synthesis of p73 protein. Consistently, we found that RPL26 directly binds to p73 3′ untranslated region (3′UTR) and that RPL26 is necessary for efficient expression of an eGFP reporter that carries p73 3′UTR. We also found that RPL26 interacts with cap-binding protein eIF4E and enhances the association of eIF4E with p73 mRNA, leading to increased p73 mRNA translation. Finally, we showed that knockdown of RPL26 promotes, whereas ectopic expression of RPL26 inhibits, cell growth in a TAp73-dependent manner. Together, our data indicate that RPL26 regulates p73 expression via two distinct mechanisms: protein stability and mRNA translation.

## INTRODUCTION

Ribosomes are necessary for protein synthesis as well as for normal cellular physiology and adaptive cellular responses to internal and external environmental stresses. Ribosome biogenesis is a highly coordinated process, including synthesis and assembly of the ribosomal RNAs (rRNAs) and ribosomal proteins [1, 2]. Impairment of ribosome biogenesis leads to ribosomal stress, resulting in aberrant cell proliferation and pathogenesis of human diseases, including cancer [3–5]. It is now clear that ribosomal stress triggers activation of p53 tumor suppressor [6–8]. In response to ribosomal stress, several ribosomal proteins, such as RPL5, RPL11, RPL23, RPL26, and RPS7, interact with MDM2 and block MDM2-mediated p53 ubiquitination and degradation, resulting in p53-dependent cell cycle arrest and apoptosis [8–16]. Recent studies suggest that there is a direct link between ribosomal proteins and p53 independent of MDM2. For example, RPL22 and RPL26 bind to p53 5′ untranslated region (5′UTR) and enhance p53 mRNA translation [7, 17–19].

p73, a p53 family tumor suppressor, is expressed as TA and ΔN isoforms. TAp73 is expressed from the P1 promoter located immediately upstream of the first exon and regulates a subset of p53 target genes as well as an unique set of target genes necessary for inducing cell cycle arrest and apoptosis [20]. Thus, TAp73 is classified as a tumor suppressor. Consistently, mice deficient in TAp73 are prone to spontaneous tumors and genomic instability [21, 22]. ΔNp73 is expressed from the P2 promoter in intron 3 and regulates a unique set of target genes that promote cell growth and survival [23, 24]. Thus, ΔNp73 has an oncogenic property.

As a p53 family protein, p73 is found to be activated in response to a variety of stresses that also activate p53 [25]. Similarly, several mechanisms that regulate p53 activity, such as phosphorylation and acetylation, are also found to regulate p73 activity [26–29]. A recent report showed that RPL5 and RPL11 regulate p73 expression by inhibition of MDM2 [30]. However, whether p73 is regulated through mRNA translation has not be explored, which prompted us to determine whether p73 mRNA translation can be regulated by a RNA-binding protein. Here, we showed that p73 expression is regulated by RPL26 via protein stability and mRNA translation.

## RESULTS

### TAp73 expression is regulated by RPL26 *via* protein stability and mRNA translation

**Figure 1:**
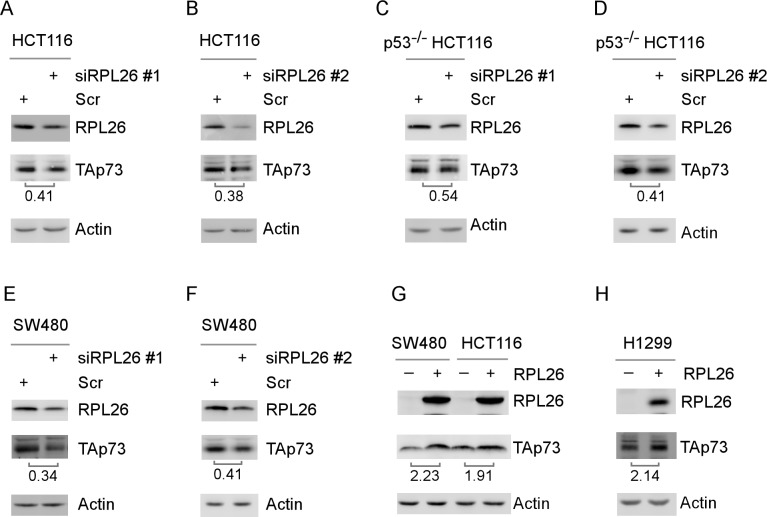
Knockdown of RPL26 decreases, whereas ectopic expression of RPL26 increases, the level of TAp73 protein **A.**-**F.** The levels of RPL26, TAp73 and actin proteins were measured in HCT116 **A.**-**B.**, p53^−/−^ HCT116 **C.**-**D.** and SW480 **E.**-**F.** cells transiently transfected with scramble siRNA, RPL26 siRNA #1 or #2 as indicated for 72 h. **G.**-**H.** The levels of RPL26, TAp73 and actin proteins were measured in SW480, HCT116 and H1299 cells transfected with an empty vector or a vector expressing RPL26 for 48 h. The data shown are representative of three independent experiments.

p73, a p53 family tumor suppressor, is tightly regulated by multiple mechanisms, including transcription and mRNA and protein stability. Since p53 mRNA translation is regulated by several RNA-binding proteins, including Rbm38 [59] and RPL26 [7], thus, there is an urgent need to determine whether p73 mRNA translation is regulated by a RNA-binding protein. Previously, we found that p73 mRNA stability but not translation is regulated by RBM38 [29]. Thus, we examined whether TAp73 expression is regulated by RPL26. We found that the level of TAp73 protein was decreased in HCT116 cells upon knockdown of RPL26 with two individual siRNAs (Figure 1A-1B). Given that p73 is a target of wild-type p53 and that p53 is regulated by RPL26 [7], we examined whether p73 is regulated by RPL26 independently of p53 in SW480 cells, which carry a mutant p53 (R273H/P309S), and p53-deficient HCT116 cells. Indeed, we found that TAp73 expression was decreased in p53^−/−^ HCT116 and SW480 cells in which RPL26 expression was knocked down by siRNAs (Figure 1C-1F). Conversely, we found that upon ectopic expression of RPL26, the levels of TAp73 protein were increased in SW480, HCT116, and p53-null H1299 cells (Figure 1G-1H).

**Figure 2:**
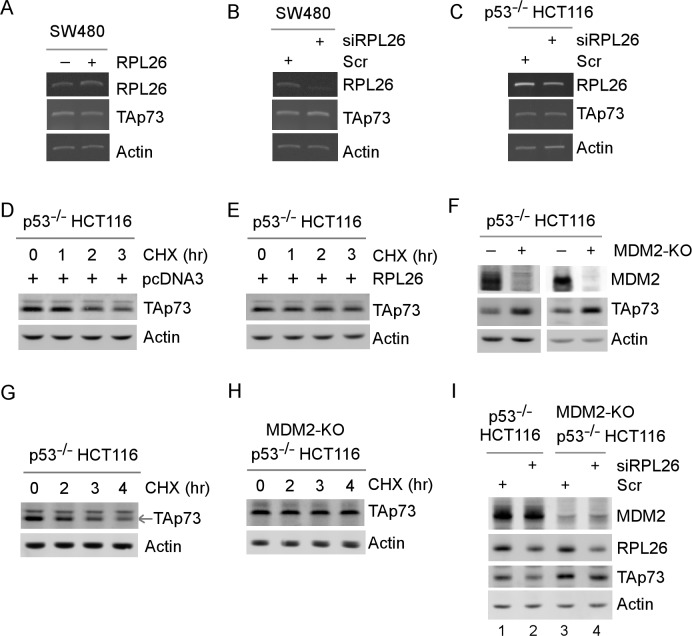
RPL26 modulates TAp73 protein stability in part *via* MDM2 **A.** The levels of RPL26, TAp73 and actin transcripts were measured in SW480 cells, which were transfected with an empty vector or a vector expressing RPL26 for 48 h. **B.**-**C.** The level of RPL26, TAp73 and actin transcripts was measured in SW480 cells **B.** or p53^−/−^ HCT116 cells **C.**, which were transfected with scrambled siRNA or siRNA against RPL26 for 72 h. **D.**-**E.** The half-life of TAp73 protein was determined in p53^−/−^ HCT116 cells, which were transfected with an empty vector **D.** or a vector expressing RPL26 **E.** for 48 h along with treatment of cycloheximide for various times. **F.** The levels of MDM2, TAp73 and actin proteins were measured in p53^−/−^ HCT116 and MDM2-knockout p53^−/−^ HCT116 cells. **G.**-**H.** The half-life of TAp73 protein was determined in p53^−/−^ HCT116 and MDM2-knockout p53^−/−^ HCT116 cells treated with cycloheximide for various times. **I.** The levels of MDM2, RPL26, TAp73 and actin proteins were measured in p53^−/−^ HCT116 and MDM2-knockout p53^−/−^ HCT116 cells, which were transfected with scramble siRNA or RPL26 siRNA as indicated for 72 h.

To determine how RPL26 regulates p73 expression, we measured p73 transcript in SW480 and p53^−/−^HCT116 cells in which RPL26 was overexpressed or knocked down. We found that the level of p73 transcript was not significantly altered (Figure 2A-2C), suggesting that RPL26 regulates p73 expression *via* a posttranscriptional mechanism. To test this, the relative stability of TAp73 protein was examined in p53^−/−^ HCT116 cells, which were transfected with an empty vector (Figure 2D) or a vector expressing HA-RPL26 (Figure 2E) for 48 h, followed by treatment with cycloheximide for various times. We found that upon ectopic expression of RPL26, TAp73 protein stability was markedly increased (Figure 2D-2E). Since RPL26 inhibits MDM2-mediated degradation of p53 *via* physical interaction with MDM2 [18], we tested whether RPL26 may regulate p73 protein stability *via* MDM2. To test this, we generated multiple p53^−/−^ HCT116 cell lines in which MDM2 was knocked out by CRISPR/cas9. Indeed, we found that in MDM2-KO p53^−/−^ HCT116 cells, the basal level of TAp73 protein was much higher than that in isogenic p53^−/−^ HCT116 cells (Figure 2F). We also found that the half-life of p73 was much longer in MDM2-KO p53^−/−^ HCT116 cells than isogenic p53^−/−^ HCT116 cells (Figure 2G-2H), suggesting that TAp73 protein stability is regulated by MDM2, consistent with a recent report that MDM2 targets p73 for degradation [31].

**Figure 3:**
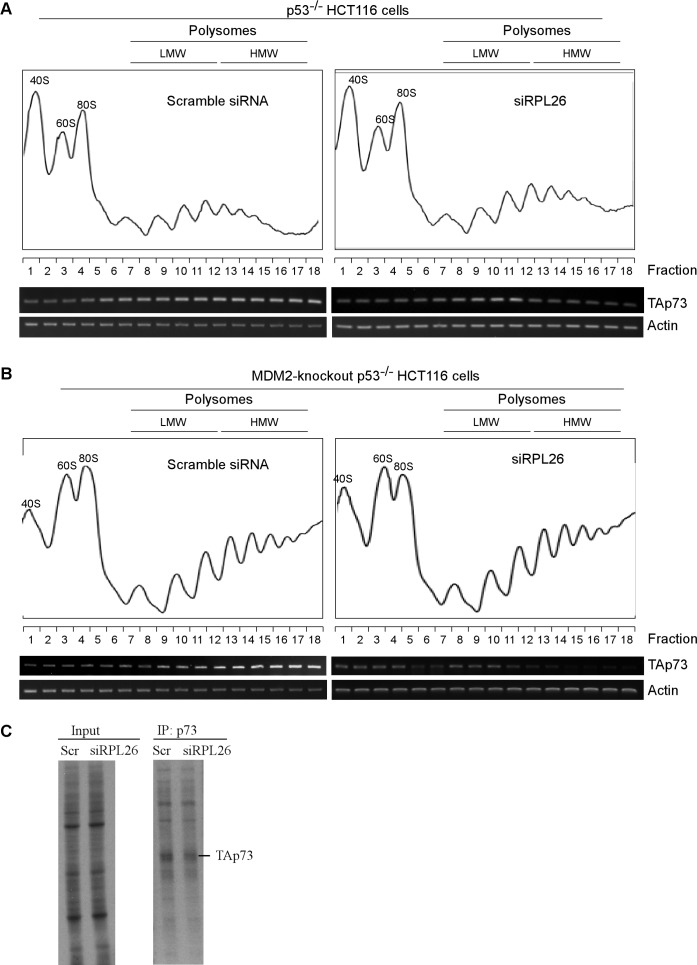
RPL26 is necessary for proper assembly of polysomes on TAp73 mRNA and mRNA translation **A.** Sucrose density gradient was used to separate polysomes from p53^−/−^ HCT116 cells transfected with scramble siRNA or siRNA against RPL26 for 72 h. The level of TAp73 and actin transcripts was measured in each fraction. **B.** The experiment was performed as in **A.** except that MDM2-knockout p53^−/−^ HCT116 cells were used. **C.** MDM2-knockout p53^−/−^ HCT116 cells were transfected with scrambled siRNA or siRNA targeting RPL26 (siRPL26) for 3 days and the level of newly synthesized TAp73 protein was measured by ^35^S-metabolic labeling.

Next, we examined whether RPL26 is still capable of regulating p73 expression in MDM2-KO p53^−/−^ HCT116 cells. Interestingly, we found that in the absence of MDM2, p73 expression was still inhibited upon knockdown of RPL26, albeit to a lesser extent than that in Mdm2-competent cells (Figure 2I, compare lanes 1 and 3 with 2 and 4, respectively). The observation suggests that RPL26 regulates p73 expression *via* other pathways in addition to MDM2. Since RPL26 is known to regulate p53 mRNA translation [7], we explored whether p73 mRNA translation is regulated by RPL26. To test this, sucrose gradient sedimentation assay was performed to examine the association of polysomes with p73 mRNA in p53^−/−^ HCT116 and MDM2-KO p53^−/−^ HCT116 cells along with or without knockdown of RPL26 (Figure 3A-3B). We found that the number of polysomes associated with TAp73 mRNA but not actin mRNA was markedly decreased upon knockdown of RPL26 in p53^−/−^ HCT116 cells (Figure 3A, TAp73 panel) as well as in MDM2-KO p53^−/−^ HCT116 cells (Figure 3B, TAp73 panel). To confirm this, the level of newly synthesized TAp73 protein was measured by ^35^S-metabolic labeling in MDM2-KO p53^−/−^ HCT116 cells transfected with scramble siRNA or siRPL26. We showed that the level of newly synthesized TAp73 protein was decreased upon knockdown of RPL26 (Figure 3C). These data suggest that RPL26 regulates p73 mRNA translation independent of MDM2.

### RPL26 regulates p73 mRNA translation *via* binding to p73 3′UTR

**Figure 4:**
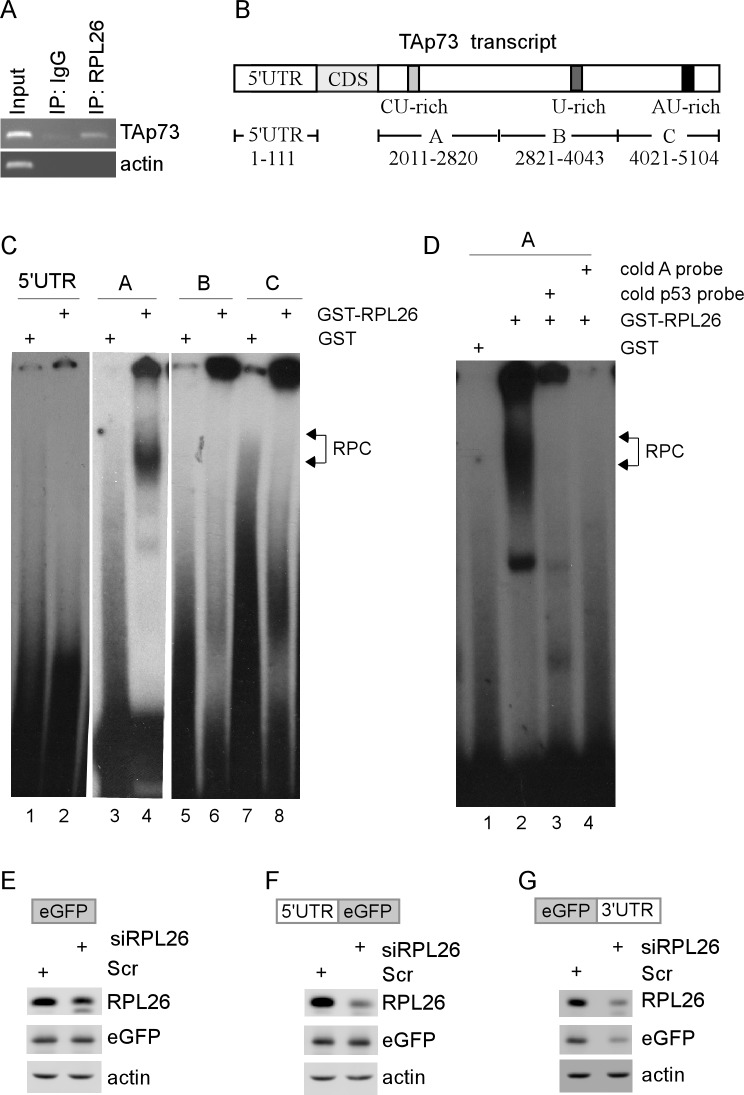
p73 3′UTR is recognized by and responsive to RPL26 **A.** RPL26 interacts with TAp73 transcript. Cell extracts from RPL26-expressing p53^−/−^ HCT116 cells were immunoprecipitated with a control IgG or anti-RPL26 antibody. The levels of transcripts for TAp73 and actin in IgG or anti-RPL26 immunocomplexes were determined by RT-PCR. **B.** Schematic presentation of TAp73 transcript and the location of probes used for REMSA. CDS represents p73 coding region. Fragments A-C cover the entire p73 3′UTR. **C.** Probes A is bound by RPL26. REMSA was performed by mixing ^32^P-labeled 5′UTR, fragment A, B, or C probe with recombinant GST or GST-RPL26 protein. The bracket indicates RNA-protein complexes (RPC). **D.** Competition assay was performed by adding an excess amount (50-fold) of a mix of cold p53 probes derived from p53 5′and 3′ UTRs, or unlabeled fragment A derived from p73 3′UTR to the reaction mix prior to incubation with the ^32^P-labeled probe A. **E.**-**G.** p73 3′ UTR is responsive to RPL26. H1299 cells were transfected with scramble siRNA (Scr) or RPL26 siRNA for 72 h, and then transfected with a vector that contains the eGFP coding region alone **E.**, the eGFP coding region plus TAp73 5′ UTR **F.**, or the eGFP coding region plus p73 3′ UTR **G.**. Whole cell lysates were collected and the levels of eGFP, RPL26, and actin were analyzed by western blot analysis.

To explore whether RPL26 directly interacts with p73 transcript to regulate mRNA translation, RNA immunoprecipitation followed by RT-PCR (RNA-ChIP) was performed with extracts from RPL26-expressing HCT116 cells. We showed that TAp73 transcript was detected in anti-RPL26 but not IgG immunoprecipitates (Figure 4A). As a control, actin transcript was not detected in anti-RPL26 and IgG immunocomplexes (Figure 4A). Next, RNA electrophoretic mobility assay (REMSA) was performed to map RPL26-binding site(s) in TAp73 transcript. Since 5′- and 3′-UTRs in a given mRNA are often recognized by a RNA-binding protein, which then regulates mRNA stability and/or translation, four RNA probes derived from TAp73 5′- and 3′-UTRs were generated and ^32^P-labeled (Figure 4B). We found that recombinant GST-fused RPL26 but not GST alone was able to form a distinct complex with probe A (Figure 4C, lanes 3-4). However, TAp73 5′ UTR and fragments B-C showed no binding with RPL26 or GST (Figure 4C, lanes 1-2 and 5-8). We would like to note that there was substantial precipitation in the wells possibly due to non-specific aggregation/precipitation [32]. Additionally, probe A-RPL26 complexes were disrupted by cold probe A or by p53 probes (Figure 4D). The p53 probes are derived from p53 5′and 3′ UTRs and known to bind to RPL26 [33].

To determine whether p73 3′ UTR is necessary and sufficient for RPL26 to regulate p73 mRNA translation, we generated three eGFP reporters that carry no p73 sequence, TAp73 5′ UTR, or p73 3′ UTR. We showed that knockdown of RPL26 led to decreased expression of eGFP protein from an eGFP reporter that carries p73 3′ UTR (Figure 4G). In contrast, RPL26 had no effect on the level of eGFP expression from an eGFP reporter that carries none or TAp73 5′ UTR (Figure 4E-4F). These results suggest that p73 3′UTR is recognized by and responsible for RPL26 to regulate p73 mRNA translation.

### RPL26 enhances the binding of eIF4E to p73 mRNA through physical interaction

**Figure 5:**
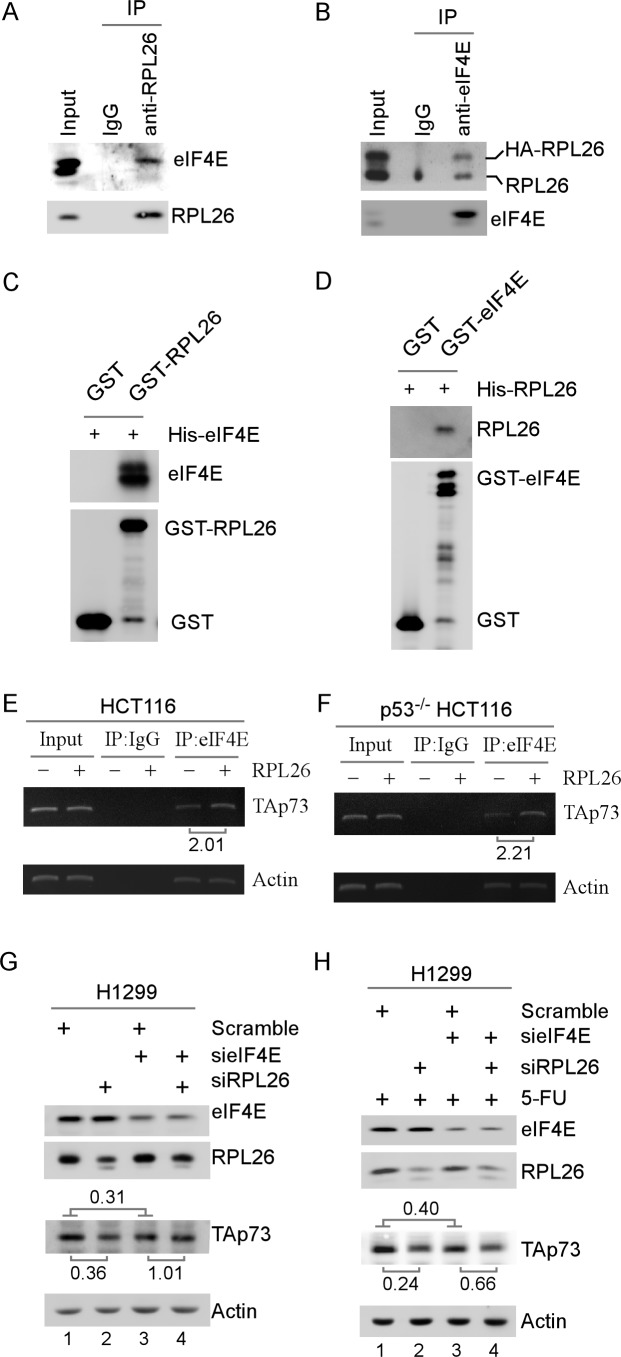
RPL26 modulates eIF4E to regulate p73 mRNA translation *via* physical interaction **A.** Lysates purified from H1299 cells were treated with RNase A and then immunoprecipitated with IgG or anti-RPL26, followed by western blot analysis with antibodies against eIF4E and HA (for HA-RPL26), respectively. **B.** Cell extracts were purified from HA-RPL26-expressing H1299 cells, treated with RNase A, and then immunoprecipitated with IgG or anti-eIF4E, followed by western blot analysis with antibodies against RPL26 and eIF4E, respectively. **C.** GST pull-down assays were performed with GST or GST-tagged RPL26 incubated with an equal amount of His-tagged eIF4E along with glutathione sepharose for 1 h. Complexes were then washed, followed by western blot analysis using antibody against histidines (anti-omini) or GST. **D.** The experiment was performed as in **C.** except that His-tagged RPL26 and GST-tagged eIF4E were used. **E.** Cell extracts from HCT116 cells, which were transfected with pcDNA3 vector or a vector expressing HA-RPL26, were immunoprecipitated with IgG or anti-eIF4E antibody. Total RNAs were purified from immunocomplexes and subjected to RT-PCR analysis to measure the levels of TAp73 and actin transcripts. The relative level of TAp73 transcript was measured by densitometry and the relative fold change was shown below each pair. The data shown are representative of three independent experiments. **F.** The experiment was performed as in **E.** except that p53^−/−^ HCT116 cells were used. **G.** The levels of eIF4E, RPL26, TAp73, and actin proteins were measured in H1299 cells transfected with siRNA against eIF4E along with scramble siRNA or siRNA against RPL26 as indicated for 72 h. The data shown are representative of three independent experiments. **H.** The experiment was performed as in **G.**, except the cells were treated with 5-FU (100 μM) for 24 h.

Translation initiation is a rate-limiting step and regulated by multiple mechanisms to control mRNA translation [34–36]. Thus, we asked whether eIF4E, the mRNA cap-binding protein and a key component of the eIF4F complex, is targeted by RPL26 to regulate p73 mRNA translation. To test this, immunoprecipitation followed by western blot analysis (IP-WB) was performed with extracts from HCT116 cells in which HA-tagged RPL26 was ectopically expressed. Prior to immunoprecipitation, cell extracts were treated with RNaseA to eliminate mRNAs, including p73 mRNA, that may bridge an interaction between RPL26 and eIF4E. We found that eIF4E was detected in RPL26-containing, but not IgG-containing, immunoprecipitates (Figure 5A). Conversely, we found that both endogenous RPL26 and HA-tagged RPL26 were detected in eIF4E-containing, but not IgG-containing, immunoprecipitates (Figure 5B). To determine whether RPL26 and eIF4E interact directly, GST pull-down assay was performed. We showed that His-tagged eIF4E bound to GST-RPL26 but not GST beads (Figure 5C). Conversely, we found that His-tagged RPL26 bound to GST-eIF4E but not GST beads (Figure 5D). Together, these data suggest that RPL26 physically interacts with eIF4E.

Next, we asked whether the interaction between eIF4E and RPL26 regulates the binding of eIF4E to TAp73 mRNA, which would promote TAp73 mRNA translation. Thus, RNA-ChIP assay was performed and showed that upon expression of HA-tagged RPL26, the relative level of TAp73 transcript associated with eIF4E was markedly increased in HCT116 cells (2.01-fold) as well as in p53^−/−^ HCT116 cells (2.21-fold) (Figure 5E-5F, TAp73 panel). As a control, the binding of eIF4E to actin mRNA was not altered by RPL26 (Figure 5E-5F, actin panel). We also showed that in H1299 cells, the level of TAp73 protein was decreased by knockdown of eIF4E at an unstressed condition (0.31-fold) (Figure 5G, compare lane 1 with lane 3) as well as at a ribosomal stress condition (treated with 5-FU) (0.40-fold) (Figure 5H, compare lane 1 with lane 3). These data are consistent with the notion that eIF4E is critical for mRNA translation [34–36]. Interestingly, we found that the effect of RPL26 knockdown to suppress p73 expression was substantially lower in eIF4E-knockdown cells than that in eIF4E-competent cells at an unstressed condition (1.01 *vs*. 0.36 fold) as well as at a ribosomal stress condition (0.66 *vs* 0.24 fold) (Figure 5G-5H). These data suggest that eIF4E is a major effector of RPL26 on p73 mRNA translation.

### RPL26 modulates growth suppression in a TAp73-dependent manner

**Figure 6:**
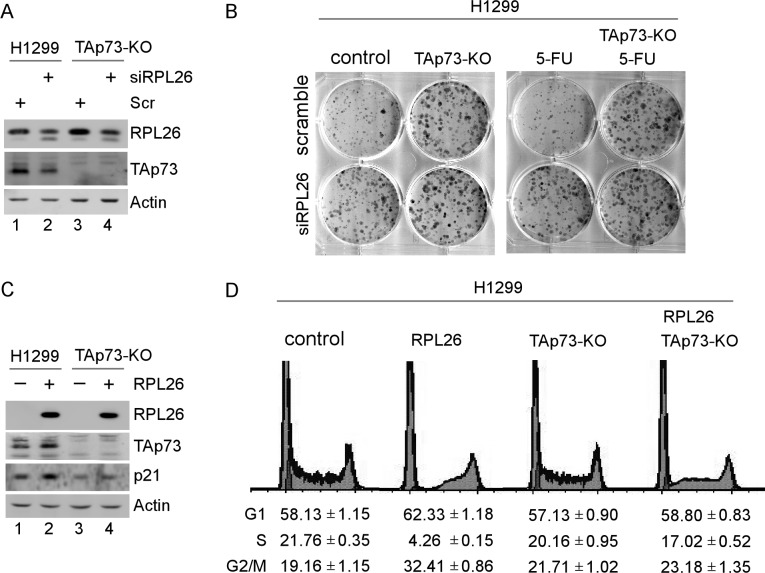
RPL26 regulates cell proliferation in a TAp73-dependent manner **A.** H1299 cells (lanes 1-2) and TAp73-knockout H1299 cells (lanes 3-4) were transfected with control siRNA or siRNA targeting RPL26 for 72 h. Cell lysates were collected and subjected to western blot analysis with antibodies against RPL26, TAp73, or actin. **B.** H1299 cells and TAp73-knockout H1299 cells were transfected with control siRNA or RPL26 siRNA for 24 h, then seeded in a 6-well plate and cultured for 2 weeks. A representative image for each treatment group was shown. **C.** H1299 cells and TAp73-knockout H1299 cells were transfected with an empty vector or a vector expressing RPL26 for 48 h. Cell lysates were collected and subjected to western blot analysis with antibodies against RPL26, TAp73, p21, or actin. **D.** H1299 cells and TAp73-knockout H1299 cells, which were transfected with an empty vector or a vector expressing RPL26 for 48 h, were collected and used for DNA histogram analysis. The content of cells in each phase of the cell cycle was calculated with FACS FlowJo software from three separate repeats and presented below each group. The data are presented as the means ± S.D.

RPL26 is known to regulate cell growth at least in part *via* p53 [7]. Like p53, TAp73 is known to play a role in growth suppression [37–39]. Thus, we examined whether the effect of RPL26 on TAp73 expression has any biological function. To avoid potential interference from the effect of p53, p53-null H1299 cell line was used to generate TAp73 knockout cell lines by CRISPR-cas9. Consistent with the studies above (Figure 1), the level of TAp73 protein was decreased upon knockdown of RPL26 (Figure 6A, compare lanes 1-2). We also showed that TAp73 protein was not detectable in TAp73-KO H1299 cells regardless of RPL26 knockdown (Figure 6A, lanes 3-4). Next, colony formation assay was performed and showed that the number of H1299 cell colonies was increased by knockdown of RPL26 (Figure 6B, compare the top and bottom wells in the first column) and by knockout of TAp73 (Figure 6B, compare the top wells in the first and second columns). This is not surprising since TAp73 and RPL26 are known to regulate cell proliferation [7, 18, 33, 37–39]. However, knockdown of RPL26 had little if any effect on the number of colonies formed by TAp73-KO H1299 cells (Figure 6B, compare the top and bottom wells in the second column). Additionally, upon treatment with 5-FU, the size of H1299 cell colonies was small (Figure 6B, the top well in the third column), consistent with early report that 5-FU is capable of suppressing cell growth [40, 41]. Interestingly, even in the presence of 5-FU treatment, the colony-forming potential for H1299 cells was increased by knockdown of RPL26 (Figure 6B, compare the top and bottom wells in the third column) as well as by knockout of TAp73 (Figure 6B, compare the top wells in the third and the fourth columns). However, the number of colonies for TAp73-KO H1299 cells treated with 5-FU was not significantly increased by knockdown of RPL26 (Figure 6B, compare the top and bottom wells in the fourth column). These observations suggest that TAp73 is primarily responsible for the effect of RPL26 on cell growth in p53-null H1299 cells.

To validate the effect of RPL26 on growth suppression in a TAp73-dependent manner, we measured the response of TAp73-competent and -deficient H1299 cells to ectopic expression of RPL26. Consistent with the above study (Figure 1), ectopic expression of RPL26 led to increased expression of TAp73 protein along with increased expression of p21, a TAp73 target (Figure 6C, compare lanes 1-2). However, in TAp73-KO H1299 cells, TAp73 was undetectable regardless of RPL26 expression (Figure 6C, compare lanes 3-4). Additionally, the effect of ectopic RPL26 on p21 expression was also diminished (Figure 6C, p21 panel, compare lanes 3-4), suggesting that TAp73 is a major activator of p21 transcription in p53-null H1299 cells. Next, DNA histogram analysis was performed and showed that upon ectopic expression of RPL26, H1299 cells underwent cell cycle arrest in G1 and G2 along with decreased number of cells in S phase (Figure 6D, compare the first two columns). Interestingly, knockout of TAp73 alone at an unstressed condition had minimal effect on the distribution of cells in various phases of the cell cycle as compared to isogenic control cells (Figure 6D, compare the first column with the third column). However, the effect of RPL26 on cell cycle arrest was mitigated by knockout of TAp73 (Figure 6D, compare the second column with the fourth column), which is consistent with the diminished effect of RPL26 on p21 expression in TAp73-KO p53^−/−^ H1299 cells (Figure 6C, p21 panel).

## DISCUSSION

Ribosomal stress leads to translocation of free ribosomal proteins from the nucleolus to the nucleoplasm wherein free ribosomal proteins increase p53 expression by interacting and then inhibiting MDM2-mediated degradation of p53 or directly activating p53 mRNA translation by binding to a stem-loop formed by p53 5′ and 3′ UTRs [42, 43]. Interestingly, alterations of ribosome biogenesis also lead to p53-independent growth suppression *via* an unknown mechanism [44, 45]. In the current study, we found that overexpression of RPL26 increases, whereas knockdown of RPL26 decreases, the level of TAp73 protein. We also found that overexpression of RPL26 increases TAp73 protein stability. Since RPL26 and other ribosomal proteins are known to increase p53 protein stability through interaction with MDM2 [8, 15, 16], we speculate that TAp73 protein stability may be similarly regulated by RPL26. Indeed, we found that knockout of MDM2 induces TAp73 expression through increase protein stability, which is consistent with some reports that MDM2 promotes p73 ubiquitination and subsequently proteosomal degradation [31, 46, 47]. However, early reports showed that MDM2 is unable to polyubiquitinate p73 for degradation [48, 49]. These conflict observations suggest that MDM2-mediated degradation of p73 is not as robust as that for p53 [46]. Other possibility is that MDM2 has to partner with other E3 ligases, such as Itch, in order to efficiently target p73 for degradation [46].

We found that in MDM2-deficient cells, RPL26 is still capable of regulating p73 expression. We also found that RPL26 is necessary for efficient assembly of polysomes on p73 mRNA. Consistently, we found that RPL26 directly binds to p73 3′UTR and that RPL26 is necessary for efficient expression of an eGFP reporter that carries p73 3′UTR. Furthermore, we found that RPL26 interacts with cap-binding protein eIF4E and enhances the association of eIF4E with p73 mRNA. Together, we hypothesize that upon binding to p73 mRNA, RPL26 interacts with eIF4E and subsequently promotes formation of translation initiation complex, leading to efficient p73 protein biosynthesis. Thus, the mechanism by which RPL26 regulates p73 mRNA translation is quite similar to that by maskin and RBM38 [50, 51], suggesting that ribosome proteins have a selective effect on mRNA translation in addition to their constitutive function as a component of ribosomes. Indeed, RPL38 regulates a subset of homeobox mRNA translation by facilitating 80S complex formation [52]. Similarly, RPS25 is found to regulate specific IRES-containing mRNA translation in yeast [53]. We would like to mention that RPL26 is capable of regulating p53 mRNA translation by binding to a stem loop formed by the complementary sequences from p53 5′ and 3′ UTRs [7, 33]. Since p73 3′UTR is recognized by and sufficient for RPL26 to regulate p73 mRNA translation, our data suggest that the mechanism by which RPL26 regulates p73 mRNA translation is different from that for p53 mRNA translation. Considering that the mechanism by which RPL26 regulates p53 mRNA translation is still uncertain, the findings in this study would provide an insight to explore p53 mRNA translation by RPL26. We would also like to mention that since MDM2 is found to modulate p53 mRNA translation [54], MDM2 may cooperate with RPL26 to modulate p73 mRNA translation.

We showed that knockdown of RPL26 decreases TAp73 expression and promotes cell proliferation in a TAp73-dependent manner. Conversely, we found that ectopic expression of RPL26 promotes TAp73 expression and inhibits cell proliferation in a TAp73-dependent manner. Our data suggest that in addition to activation of p53, p73 expression is regulated under a ribosomal stress, such as treatment with 5-FU, *via* accumulation of ribosome-free ribosomal proteins, including RPL26. Thus, future studies are warranted to explore how p53 and p73 are coordinately regulated by ribosomal proteins under a ribosomal stress.

## MATERIALS AND METHODS

### Reagents

Anti-GST and anti-histidine were purchased from Santa Cruz Biotechnology (Santa Cruz, CA). Anti-RPL26 and anti-TAp73 were purchased from Bethyl Laboratories (Montgomery, TX). Anti-HA was purchased from Covance (San Diego, CA). Anti-actin, proteinase inhibitor cocktail, RNase A, and protein A/G beads were purchased from Sigma (St. Louis, MO). The Iscript cDNA synthesis kit was purchased from Bio-Rad Laboratories (Irvine, CA). α-^32^P-UTP was purchased from PerkinElmer (Waltham, MA). The Ni-NTA agarose beads were purchased from Biontex (Germany). The glutathione sepharose beads were purchased from Macherey-Nagel (Germany).

### Plasmids

To generate HA-tagged RPL26 expression vector, RPL26 cDNA was amplified with forward primer 5′ ATG AAG TTT AAT CCC TTT GTG AC 3′ and reverse primer 5′ TTA TTC CTG CAT CTT CTC AAT G 3′. The PCR product was inserted into a pcDNA3 vector through E*coR*I and X*ho*I sites and confirmed by sequencing. To generate vectors expressing GST- or HIS-tagged RPL26, the PCR product was inserted into pGEX vector or pcDNA3.1/HisB vector through E*coRI* and X*hoI* sites and confirmed by sequencing. HIS- or GST-tagged eIF4E expression vectors were used as previously described [55].

To generate an eGFP expression vector carrying p73 5′ or 3′UTR, DNA fragment containing p73 5′ or 3′UTR was amplified using cDNA from H1299 cells as template. The primers to amplify p73 5′UTR are forward primer 5′- AAA AAG CTT ACT AGT CGC AGC GAA ACC GGG GCC CGC -3′ and reverse primer 5′-AAA GGA TCC GCC CTG GGC CTC CTA CC-3′. The primers to amplify p73 3′UTR are forward primer 5′-AAA CTC GAG GCC CAT CAA GGA GGA GTT CA-3′ and reverse primer 5′-AAA TCT AGA AAT CCC CAC TGA AAC ACA GC-3′. The PCR products were digested with *Hind*III and *BamH*I for TAp73-5′UTR or *Xho*I and *Xba*I for p73-3′UTR and cloned into pcDNA3/eGFP vector as previously described [56]. The vectors were designated as 5′UTR-eGFP and eGFP-3′UTR.

### Cell culture and generation of knockout cell lines

SW480, HCT116, p53^−/−^ HCT116, and H1299 cells were cultured in DMEM (Invitrogen) supplemented with 10% fetal bovine serum (Hyclone). MDM2 knockout cell lines were generated by CRISPR-cas9-mediated genome editing technology. sgRNAs targeting *MDM2* were designed using the CRISPR design tool (http://tools.genome-engineering.org) and cloned into the BbsI sites of CRISPR vector pSpCas9(BB)-2A-Puro. Two gRNAs were used: gRNA #1 AGG GTC TCT TGT TCC GAA GC and gRNA #2 GTG GTT ACA GCA CCA TCA GT. The gRNA expression vector was transfected to p53^−/−^ HCT116 cells. The MDM2-KO lines were selected with puromycin and confirmed to carry a deletion in the gene encoding Mdm2 by sequencing. TAp73-KO H1299 cell lines were similarly genarated with two gRNAs: 5′-CTT CCC CAC GCC GGC CTC CGA GG-3′ and 5′-TCA AAC GTG GTG CCC CCA TCA GG-3′.

### Western blot analysis, immunoprecipitation and GST-pull down assay

Cells were cultured at various conditions and whole cell lysates were prepared by using 2X SDS sample buffer. Whole cell lysates were separated in 8~12% SDS-PAGE, transferred to a nitrocellulose membrane, and incubated with primary and secondary antibodies, followed by enhanced chemiluminescent detection.

Immunoprecipitation assay was performed as previously described [57]. Briefly, cells were lysed in 0.2 % Triton lysis buffer (25 mM Tris [pH 7.4], 25 mM NaCl, 0.2 % Triton X-100) supplemented with the proteinase inhibitor cocktail (100 μg/ml), followed by incubation with 1 μg of antibody or control IgG. The immunocomplexes were precipitated by protein A/G beads and then subjected to western blot analysis.

For GST-pull down assay, the recombinant His- and GST-tagged proteins were expressed in bacteria BL21 and purified by Ni-NTA and glutathione sepharose beads, respectively. 500 ng of recombinant His-tagged proteins and 500 ng of recombinant GST-tagged proteins were incubated in E1A binding buffer (50 mM HEPES, pH 7.6, 50 mM NaCl, 5 mM EDTA, 0.1% Nonidet P-40, and 10% glycerol) for 2 h at 4°C, followed by precipitation with glutathione-sepharose beads. Beads were washed and re-suspended in 2x SDS loading buffer and subjected to western blot analysis.

### ^35^S metabolic labeling and Immunoprecipitation

These assays were performed as previously described [57, 58].

### Probe labeling and RNA Electrophoretic Mobility Shift Assay (REMSA)

TAp73 5′UTR and various regions in p73 3′UTR were PCR amplified using primers containing T7 promoter sequence (5′-GGA TCC TAA TAC GAC TCA CTA TAG GGA G-3′). All probes were labeled by *in vitro* transcription using a DNA fragment containing T7 promoter and various region of p73 5′ or 3′UTR. Briefly, 500 ng of purified PCR product was incubated with 50 μCi of α-^32^P-UTP, 0.5 mM each of NTP (A, G, C), and 20 unit of T7 RNA polymerase (Ambion) in 20 μl of reaction at 37°C for 1 h, followed by treatment with DNase I (1 unit) for 15 min. The reaction mixture was purified by sephadex G-50 column to remove unlabeled free nucleotides and the radioactivity of probes was measured by a scintillation counter. REMSA was performed with 200 nM RPL26 recombinant protein, 100 μg/ml of yeast tRNA, and 50,000 CPM ^32^P-labeled RNA probe in 20 μl of reaction buffer (10 mM Tris-Cl, pH 8.0, 25 mM KCl, 10 mM MgCl2, 1 mM DTT) at 25°C for 25 min. RNA/protein complexes were digested by adding 100 U RNase T1 at 37°C for 15 min and then separated in 7% of native PAGE gel. RNA-protein complexes were visualized by autoradiography. To test the specificity of RPL26 binding to p73 3′UTR, competition assay was performed by adding an excess amount of unlabeled cold fragment A probe from p73 3′UTR or cold p53 probe into the reaction mixture prior to addition of α-^32^P-labeled probe. The p53 probe are derived from p53 5′and 3′ UTRs, which were generated as previous described [59].

### Protein half-life assay

Cells were treated with 50 μg/ml of cycloheximide to inhibit de novo protein synthesis for various times. The relative levels of TAp73 protein were quantified by western blotting and normalized by levels of actin protein.

### Polysome profile analysis

p53^−/−^ HCT116 cells were transfected with scramble or siRNA targeting RPL26 for 72 h. The cells were then treated with 100 μg/mL cycloheximide for 30 min and lysed in a buffer containing 0.5% NP40, 0.1 M NaCl, 10 mM MgCl2, 2 mM DTT, 50 mM Tris-HCl (pH 7.5), 200 U/mL SUPERase·In RNase inhibitor, 100 μg/mL cycloheximide, and 200 μg/mL heparin. Nuclei were precipitated at 12,000g for 10 min. The resulting supernatants were layered on a 15% to 45% (w/v) sucrose gradient containing 0.15 M NaCl, 5 mM MgCl2, and 25 mM Tris-HCl (pH 7.5), and centrifuged in a SW40 rotor (Beckman Coulter) at 35,000 rpm for 150 min. RNA-protein complexes in the gradients were fractioned by ISCO fractionator with 254 nm UV detector. Total RNAs were extracted from RNA-protein complexes with phenol-chloroform-isoamyl alcohol and recovered by ethanol precipitation. One microgram of total RNAs from each fraction was used for RT-PCR to detect TAp73 and actin transcripts.

### RNA interference

Scramble siRNA (5′ GCA GUG UCU CCA CGU ACU A dTdT 3′), siRNA against RPL26 #1 (5′ CCG AAA GGA UGA UGA AGU U dTdT 3′), and siRNA against RPL26 #2 (5′ CAC AUU CGA AGG AAG AUU A dTdT 3′) were purchased from Dharmacon (Chicago, IL). For siRNA transfection, siLentFectTM Lipid Reagent (Bio-Rad) was used according to the user's manual.

### RNA isolation and RT-PCR analysis

Total RNA was isolated with Trizol reagent. RT-PCR was performed with the Iscript cDNA synthesis kit (Bio-Rad Laboratories) according to the manufacturer's instruction. The primers used to amplify human actin were forward primer 5′ CTG AAG TAC CCC ATC GAG CAC GGC A 3′ and reverse primer 5′ GGA TAG CAC AGC CTG GAT AGC AAC G 3′. The primers for human TAp73 were forward primer 5′ CAG ACA GCA CCT ACT TCG AC 3′ and reverse primer 5′ CTG CTC ATC TGG TCC ATG G 3′. The primers for human RPL26 were forward primer 5′ CGA TCC ATG CCC ATC CGA AA 3′ and reverse primer 5′ TGC CTA CGT GGA CAG TTG TG 3′.

### RNA-immunoprecipitation (RNA-IP)

RNA-IP was carried out as previously described [60, 61). Briefly, cells (2 × 10^7^) were lysed with 1 ml of lysis buffer (10 mM HEPES, pH7.0, 100 mM KCl, 10 mM MgCl_2_, 0.5% NP-40, 1 mM DTT) supplemented with RiboLock Ribonuclease inhibitor (Fermentas) for 30 min on ice, and cell lysates were collected by centrifugation (13,000 rpm at 4°C for10 min). The RNA-protein immunocomplexes were incubated with 2 μg of anti-RPL26 or isotype control IgG at 4°C for 4 h and brought down by protein G beads. RT-PCR analysis was carried out to examine the relative levels of various RNAs purified from RNA-protein complexes.

### Colony formation assay

1,000 cells/well were plated in a six-well plate. After 15 days, colonies were fixed with methanol, stained with crystal violet, and then counted.

### DNA histogram analysis

H1299 cells and TAp73-knockout H1299 cells were transfected with an empty vector or a vector expressing RPL26 for 48 h. Both floating dead cells in the medium and live cells on the plate were collected and fixed with 70% ethanol for 24 h at 4°C. The fixed cells were centrifuged and resuspended in 0.3 ml of PBS containing 50 μg/ml each of RNase A and propidium iodide (PI). The stained cells were analyzed using a fluorescence activated cell sorter. The percentage of cells in sub-G1, G1, S, and G2-M phases were determined using the FACS FlowJo software (Treestar, San Carlos, CA).
